# An Epidemiological Model of the Effects of Insecticide-Treated Bed Nets on Malaria Transmission

**DOI:** 10.1371/journal.pone.0144173

**Published:** 2015-12-04

**Authors:** Philip L. G. Birget, Jacob C. Koella

**Affiliations:** 1 Silwood Park, Imperial College London, Ascot, United Kingdom; 2 Institute of Biology, Université de Neuchâtel, Neuchâtel, Switzerland; Instituto de Higiene e Medicina Tropical, PORTUGAL

## Abstract

Insecticide-treated bed nets (ITNs) have become a central tool for malaria control because they provide personal and community-wide protection through their repellent and insecticidal properties. Here we propose a model that allows to assess the relative importance of those two effects in different epidemiological contexts and we show that these two levels of protection may oppose each other. On the one hand, repellency offers personal protection to the users of ITNs. The repellent action, however, is a two-edged sword, for it diverts infectious mosquitoes to non-users, thereby increasing their risk. Furthermore, with increasing ITN coverage, the personal protection effect of repellency decreases as mosquitoes are forced to perform multiple feeding attempts even on ITN users. On the other hand, the insecticidal property, which offers community-wide protection by killing mosquitoes, requires that mosquitoes contact the insecticide on the ITN and is thus counteracted by the repellency. Our model confirms that ITNs are an effective intervention method by reducing total malaria prevalence in the population, but that there is a conflict between personal protection, offered by repellency, and community-wide protection, which relies on the ITN’s insecticidal properties. Crucially, the model suggests that weak repellency allows disease elimination at lower ITN coverage levels.

## Introduction

Insecticide-treated bed nets (ITNs) are among the most important and cost-effective intervention measures against malaria relying on three main mechanisms: 1) the nets create a physical barrier between the human and the mosquito vector, 2) the insecticide used to treat the bed net repels mosquitoes (“excito-repellency” or “deterrence”, simply referred to as repellency in this paper), thus increasing the personal protection offered by the net, and 3) if a mosquito fails to be repelled, it will often rest on the bed net after biting, and may then be killed by contacting the insecticide. For mosquitoes with some degree of zoophily (also known as zoophagy or animal feeding), ITNs also provide protection by diverting mosquitoes to non-human hosts [1–3]. In addition, ITNs may increase the host searching time of a mosquito, which increases the duration of the gonotrophic cycle and thus the risk that the mosquito dies before obtaining its blood-meal [4]. ITNs thus offer a mix of personal protection—blocking the bites of mosquitoes, thereby reducing the transmission from mosquitoes to humans—and community protection—reducing the longevity of mosquitoes and therefore the prevalence of sporozoites, the infectious stage of malaria, in mosquitoes.

The personal protection offered by insecticide-treated bed nets has been documented in many studies e.g [5–8]. In a large randomised control trial, for example, Gimnig et al [5] found up to 95% lower resting densities of mosquitoes in houses with nets than in houses without nets, and mosquitoes were less likely to carry sporozoites. Comparisons before and after extensive bed net coverage showed similar results [9–11]. ITNs also have a good record of reducing the intensity of transmission within the whole community i.e. not only among ITN-users but also among non-users. Hawley et al [12], for example, found reduced disease incidence up to 300m around a house where ITNs are used, and [13] report a 4.2 fold reduction of the entomological inoculation rate (EIR) experienced by unprotected people with a coverage of 75% of untreated nets and an 18-fold reduction if those nets are treated with insecticides.

Although these field studies provide valuable information about the success of ITNs for malaria control, it is not clear which aspect of the ITN—its physical barrier, its insecticidal effect or its excito-repellency—is the most important characteristic in reducing malaria transmission. Though many field studies show a positive effect of ITN coverage even to non-users, the possibility remains that, at some levels of coverage and some epidemiological settings, ITNs divert mosquitoes to unprotected people in a way that increases their risk, making ITNs an ethically challenging intervention [1, 14–17].

Several studies have attempted to fill that gap by proposing models that predict the impact of ITNs on disease transmission. Chitnis et al [2] developed a mathematical formalisation of malaria model where mosquitoes are allowed to obtain blood from a diverse host population, which is essential if we want to model the effect of ITNs as we need at least two categories of humans hosts, ITN-users and non-ITN users. In a numerical simulation applied to ITNs, they find that ITNs have a community-wide positive effect. Similarly, Killeen and colleagues used a description of the mosquito’s feeding behaviour to calculate the relative exposure of protected compared to unprotected hosts and the effect of ITNs on the EIR [3, 18–20]. An extension of these models allowed to disentangle the protective effects of the various properties of the ITN, namely their insecticidal effect (toxicity) and repellency [17]. The authors showed that repellency may indeed erode community-wide protection offered by high ITN coverage. This finding is confirmed by Gu et al. [16], who developed an individual-based model of mosquitoes feeding in a village surrounded by breeding areas and calculated the effect of various coverage by ITNs on the mosquito dynamics and human prevalence. The conclusions of the paper were that the effectiveness of the nets was most sensitive to the insecticidal effect of the insecticide and that a strong repellent effect of impregnated nets can lead to a greater risk for people who do not use bed nets. Finally, LeMenach et al [4], who described the feeding success and survival of mosquitoes in a gonotrophic cycle by a mathematical dissection of their feeding behaviour, make the point that one of the reasons for the effectiveness of ITNs is that zoophilic mosquitoes are diverted to non-human hosts. Thus, whether repellency offers community-wide protection or not crucially depends on the feeding preferences of the vector (see [1] for a detailed review).

While these studies confirm that ITNs generally have a protective effect and, indeed, that repellency can increase the risk among unprotected individuals, they generally lack a solid integration of the epidemiological dynamics of infections in humans and mosquitoes by assuming fixed values of infectivity from humans to mosquitoes. Most of the models described above calculate the effect of ITNs on the entomological inoculation rate (EIR), a measure of the intensity of transmission. Though there are models of how to deduce the actual prevalence of malaria in the human population from the EIR [21], it would be desirable to directly model prevalence (also called parasite rate) in infected and uninfected people, especially since the latter has been an important quantity for intervention method decision making [21, 22]. The coupling of malaria infection dynamics of humans with those of mosquitoes also allows more precise modelling of herd effects, which is a crucial component of the ITN intervention. In this study we therefore extend the classical Ross-Macdonald model for malaria transmission [23–25], see [26] for a recent review) to describe malaria transmission and its prevalence in ITN-protected and unprotected people, and combine the model with the vector’s behavioural parameters that are derived from a mosquito feeding cycle.

## Materials and Methods

**Fig 1:**
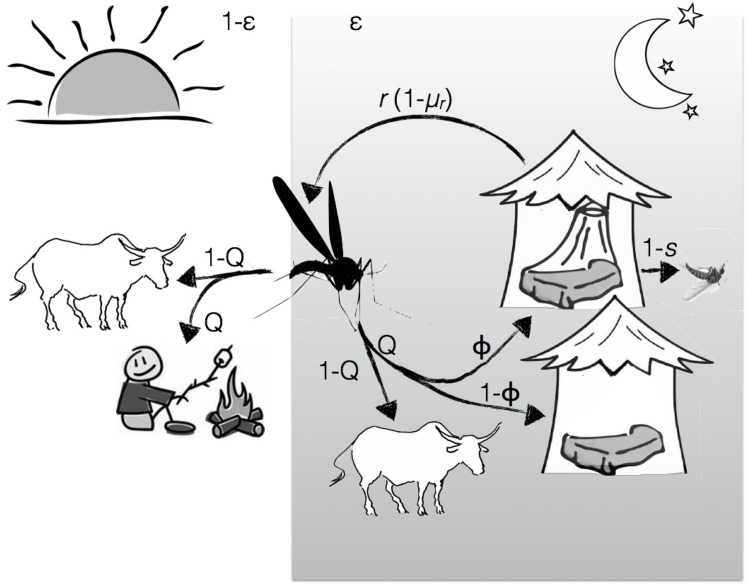
Host searching cycle of a mosquito. A mosquito bites indoors with probability *ϵ* (for night-active and highly anthropophilic mosquitoes, this happens mainly at night) and takes a bite outdoors with probability 1 − *ϵ*. A mosquito then bites humans with a probability *Q*. If biting indoors, it will enter a house where a person sleeps under a bed net with a probability *ϕ* (the ITN coverage) or a house with an unprotected person with a probability 1 − *ϕ*. If the person is protected, the mosquito is repelled by the insecticide (or mechanically blocked by the net) with a probability *r*; if it is not repelled, it takes its bite and escapes with probability *s* or it is killed by the insecticide on the net with probability (1 − *s*). If a mosquito is repelled by a bed net, it leaves the house and continues to search for a host. There is a mortality cost *μ*_*r*_ associated with each repellency event. We assume that a mosquito will always land a successful bite on unprotected people and and on animals, whereas the feeding success on protected people depends on *r* and *s*. The host search happens once per mosquito gonotrophic cycle, i.e. once every three days (see Table 1).

Our epidemiological model combines epidemiological theory [25] with equations describing the mosquito’s feeding cycle and its behavioural response to ITNs (Fig 1).

### Feeding cycle

We extended the approach described by LeMenach et al [4] to calculate the proportion of mosquitoes that bite ITN-users (a proportion *ϕ* of the population) and non-users (proportion 1 − *ϕ*). We distinguish two stages of the biting attempts: the probability of initiating a bite and probing on a human (which suffices for the transmission of malaria from mosquitoes to humans) and the probability of completing the bite and surviving possible contact with the insecticide (which is required for the transmission from human to mosquitoes). To calculate these probabilities, we assumed that host-seeking mosquitoes target humans with a probability *Q* and other animals with probability 1 − *Q*, and that of the mosquitoes targeting humans, a proportion *ϵ* are endophilic, i.e. bite at a time when humans are sleeping indoors, while a proportion 1 − *ϵ* are exophilic (note that in our model endophily is irrelevant for mosquitoes targeting animals). If the indoor-host is protected by an ITN, the mosquito is repelled and starts a new host search with probability *r*. Note that the repellency parameter includes both, repellency caused by volatiles of the insecticide as well as repellency due to the sheer physical feature of the net. If it is not repelled (probability 1 − *r*), it overcomes the mechanical protection offered by the net to blood-feeds, but is killed by the insecticide with probability 1 − *s*. Thus, a mosquito can initiate a bite on an ITN-user in two ways. First, it can target ITN-users during the time when they are still outdoors. The probability of this event is

$$\begin{array}{c} H_{p,o}=Q\left(1-\epsilon \right)\phi \end{array}$$ (1)

Second, if it bites indoors (at a time when people are sleeping), it can target an ITN-user during its first biting attempt, during its second attempt (having been repelled once), during its third attempt (having been repelled twice), etc. The probability of biting an ITN-user after a single attempt is *Qϕ*(1 − *r*); if each additional search of a host brings with it the risk *μ*_*r*_ of dying, the probability of having been repelled *n* times is (*Qϕr*(1 − *μ*_*r*_))^*n*^. Thus, the probability that a mosquito initiates a bite on an ITN-user sleeping indoors is

$$\begin{array}{ccc} H_{p,i} & = & Q\epsilon \phi \left(1-r\right)\sum_{n=0}^{\infty }{\left[Q\phi r\left(1-\mu_{r}\right)\right]}^{n}\\ & = & \frac{Q\epsilon \phi (1-r)}{1-Q\phi r(1-\mu_{r})} \end{array}$$ (2)

The probability that a mosquito bites an ITN-user (indoors or outdoors), *H*_*p*_, is the sum of Eqs (1) and (2):

$$\begin{array}{c} H_{p}=Q[1-(1-\frac{\left(1-r\right)}{1-Q\phi r(1-\mu_{r})})\epsilon ]\phi \end{array}$$ (3)

Similarly, the probability that a mosquito bites an unprotected person (indoors or outdoors) is

$$\begin{array}{c} H_{u}=Q[1-(1-\frac{1}{1-Q\phi r(1-\mu_{r})})\epsilon ]\left(1-\phi \right) \end{array}$$ (4)

These ideas can be extended to calculate the probabilities that mosquitoes survive their biting attempts on ITN-users, *P*_*p*_, and non-users, *P*_*u*_. As we standardized the equations by letting all mosquitoes survive their biting attempts on unprotected hosts, *P*_*u*_ = *H*_*u*_; the probability of surviving a biting attempt on a protected host is

$$\begin{array}{c} P_{p}=Q[1-(1-\frac{(1-r)s}{1-Q\phi r(1-\mu_{r})})\epsilon ]\phi \end{array}$$ (5)

### Human dynamics

We modified the system of differential equations describing the epidemiology of malaria [25] by writing separate equations for the prevalence of disease in protected people, *y*_*p*_, and in unprotected people, *y*_*u*_:

$$\begin{array}{c} {\dot{y}}_{p}=m\;w\;a\;\frac{H_{p}}{\phi }\left(1-y_{p}\right)-\rho \;y_{p} \end{array}$$ (6)

$$\begin{array}{c} {\dot{y}}_{u}=m\;w\;a\;\frac{H_{u}}{1-\phi }\left(1-y_{u}\right)-\rho \;y_{u} \end{array}$$ (7)

where *m* is the the number of mosquitoes per person, *w* is the proportion of mosquitoes that are infectious (i.e. that carry sporozoites in their salivary glands), *a* is the biting rate of the mosquitoes on humans, and *ρ* is the recovery rate from malaria. (Note that *H*_*p*_ and *H*_*u*_ are the probabilities within the total human population that a mosquito bites a protected or an unprotected person, respectively; to get the probabilities within the protected or unprotected sub-populations, we must divide the former probabilities by *ϕ* or 1 − *ϕ*.)

### Mosquito dynamics

To simplify, we assumed that each infected person is infectious to mosquitoes. We calculated the inoculation rate of mosquitoes by averaging the probabilities that a mosquito successfully feeds on a protected person (*P*_*p*_) or on an unprotected person (*P*_*p*_): *A* = *P*_*p*_ *y*_*p*_ + *P*_*u*_ *y*_*u*_

We calculated the mosquito’s mortality from the feeding cycle. According to our assumptions, mosquitoes die if their attempt at blood-feeding is not successful. The probability of completing a blood-meal—whether on a protected human, an unprotected humans or an animal—is the sum of the probabilities of success during a single attempt, accounted by the probability that the mosquito could have landed a successful bite after *n* repellency events:

$$\begin{array}{ccc} S_{b} & = & \left(\left(1-Q\right)+Q\left(1-\epsilon \right)+Q\epsilon \left(1-\phi \right)+Q\epsilon \phi \left(1-r\right)s\right)\sum_{n=0}^{\infty }{\left[Q\epsilon \phi r\left(1-\mu_{r}\right)\right]}^{n}\\ & = & \frac{1-Q\epsilon \phi (1-(1-r)s)}{1-Q\epsilon \phi r(1-\mu_{r})} \end{array}$$ (8)

Once fed, the mosquito must survive through the duration of its gonotrophic cycle (i.e. the time it takes to develop and lay its eggs) before it starts a new feeding attempt. The probability of feeding-independent mortality during the gonotrophic cycle is *μ*_*τ*_ = 1 − (1 − *μ*_0_)^*τ*^, where *μ*_0_ is the feeding-independent daily mortality and *τ* is the duration of the gonotrophic cycle (note that, in contrast to [4], we assume that the gonotrophic cycle is not prolonged by repeated host searches. This is a good approximation unless each search for a host lasts a long time or coverage is close to 100% so many searches are necessary). The probability of surviving a gonotrophic cycle is the combination of feeding-related and feeding-independent mortality:

$$\begin{array}{c} S=\left(1-\mu_{\tau }\right)\frac{1-Q\epsilon \phi (1-(1-r)s)}{1-Q\phi r(1-\mu_{r})} \end{array}$$ (9)

giving the daily mortality rate

$$\begin{array}{c} \mu =-ln(1-{\left(1-S\right)}^{1/\tau } \end{array}$$ (10)

Following earlier approaches [25], we can then describe the dynamics of the proportion of latent (*v*) and infectious (*w*) mosquitoes as:

$$\begin{array}{c} \dot{v}=a\left(1-v-w\right)A-a\hat{A}\left(1-\hat{v}-\hat{w}\right)S^{T/\tau }-\mu v \end{array}$$ (11)

$$\begin{array}{c} \dot{w}=a\hat{A}\left(1-\hat{v}-\hat{w}\right)S^{T/\tau }-\mu w \end{array}$$ (12)

where the incubation period of malaria in mosquitoes is *T* days and where $\hat{v}$, $\hat{w}$ are the number of latent and infectious mosquitoes and $\hat{A}$ the infectious reservoir *T* days earlier. As the epidemiological dynamics in the mosquitoes are much more rapid than those of the humans, we considered them to be at equilibrium relative to the humans and therefore set $\dot{v}=0$ and $\dot{w}=0$. Thus we obtained an expression for *w* as a function of the prevalences of protected and unprotected people in *A*:

$$\begin{array}{c} w=\frac{aAS^{T/\tau }}{aA+\mu } \end{array}$$ (13)

We found the equilibrium prevalences by calculating the equilibria of Eqs (6) and (7) with the function stode of the R-package rootSolve [27]. Parameter values were obtained from published studies of the highly anthropophilic *Anopheles gambiae* species complex and *Plasmodium falciparum* (Table 1). Note that the parameter “density of mosquitoes” includes parameters that are not explicitly given in the equations, e.g. the probabilities of infection and variabilities of parameters; its value was therefore chosen to give a reasonable description of the epidemiology rather than to reflect observed densities of mosquitoes.

**Table 1:** Parameters and variables. All parameters were set to their typical values unless explicitly mentioned.

Parameter	Explanation	Typical value	Reference
*ϕ*	ITN coverage	0.5	
*m*	mosquitoes per person	1	
*a*	biting rate (per day)	0.33	[28]
*ρ*	recovery rate from malaria (per day)	0.01	[24]
*Q*	probability of feeding on humans	0.95
*ϵ*	probability of indoor feeding	0.9	[29]
*r*	probability of repellency	0.6-0.9	[30]
*s*	survival after feeding	0.16	[30]
*μ*_0_	background mortality of mosquitoes (per day)	0.1	[31]
*μ*_*r*_	mortality during host searching (per search)	0.03	
*T*	time for sporozoite development (days)	10.3	[32]
*τ*	duration of gonotrophic cycle	3 days	
**Variables**			
*y*_*p*_	prevalence of malaria in protected individuals		
*y*_*u*_	prevalence of malaria in unprotected individuals		
*v*	number of latently infected mosquitoes		
*w*	number of infectious mosquitoes		

## Results

**Fig 2:**
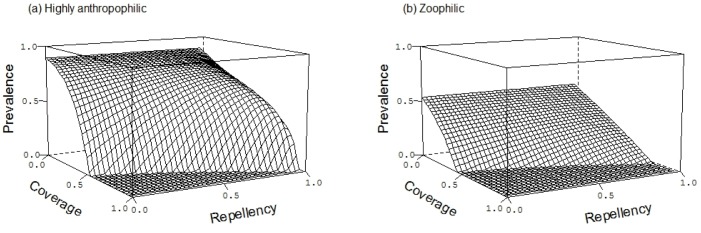
The effect of bed net coverage (*ϕ*) and repellency (*r*) on malaria prevalence. Panel (a) shows a situation with highly anthropophilic mosquitoes (*Q* = 0.95; panel (b) with zoophilic mosquitoes (*Q* = 0.3). Other parameters are given in Table 1.

We find that increased coverage of ITNs decreases malaria prevalence through a combination of the personal protection given by the repellency of the insecticide and the community protection given by its insecticidal action (Fig 2). Whether it is personal protection or community protection that is more relevant depends on the context defined by the details of the parameters.

**Fig 3:**
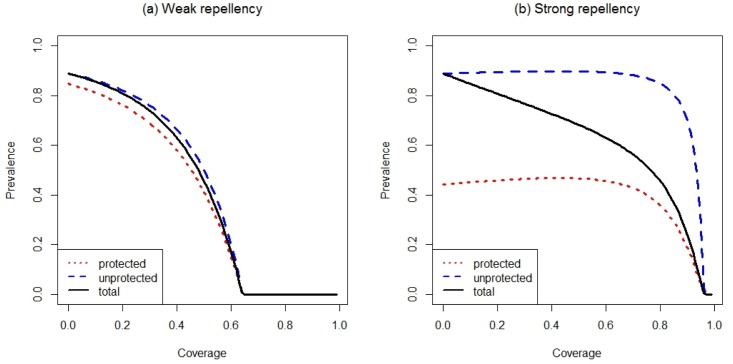
The effect of coverage on malaria prevalence at the epidemiological equilibrium. Prevalence in unprotected people is shown by the dashed line, in protected people by the dotted line, and the population as a whole is represented by the solid line. In panel (a) repellency is *r* = 0.3, in panel (b) *r* = 0.9. Other parameters are given in Table 1.

If repellency is weak, personal protection against mosquito bites is low, so that most of the impact on prevalence is due to the insecticidal action of the bed nets. As coverage increases, so does the number of mosquitoes killed by the insecticide, thus decreasing transmission and prevalence in protected and, through a herd effect, unprotected people (Fig 3a). If repellency is stronger, the bed nets provide more personal protection but fewer mosquitoes contact the insecticide and die. This leads to a greater difference in prevalence between protected and unprotected people who are infected. Furthermore, as coverage increases, more mosquitoes are diverted to unprotected people, which increases the risk of the unprotected people (Fig 3b). More surprisingly, as coverage of strongly repellent nets increases, so does the prevalence in *protected* people (Fig 3b). The reason is that repelled mosquitoes are not only diverted to unprotected humans and animals as some of the repelled mosquitoes will attempt to bite protected individuals. Some of these attempts will also be successful if repellency is not complete. Thus, although people using an ITN obtain personal protection, the fact that their neighbors also use ITNs makes this protection less effective.

However, it is important to bear in mind that, although prevalence of protected and unprotected may increase with ITN coverage, total prevalence still decreases because increasing coverage, by definition, means moving people from the unprotected to the protected category with the latter facing a substantially smaller risk of receiving an infectious bite than the former.

**Fig 4:**
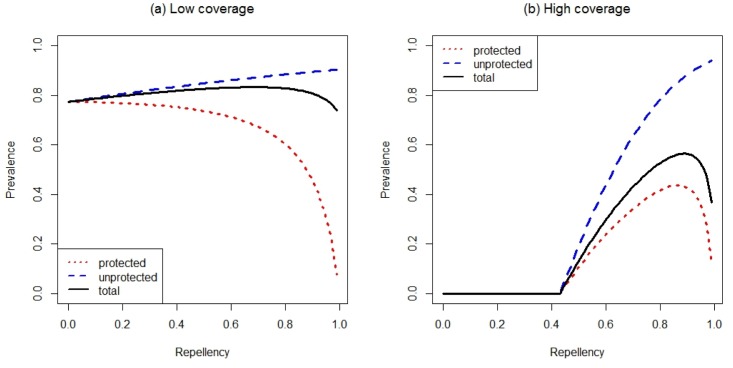
The effect of repellency on malaria prevalence at the epidemiological equilibrium. Unprotected people are represented by the dashed line, protected people by the dotted line, and the population as a whole by the solid line. In panel (a) coverage is *ϕ* = 0.2, in panel (b) *ϕ* = 0.7. Other parameters are given in Table 1.

Similar arguments explain why increasing repellency *increases* prevalence and increases the coverage required to eliminate the parasite (Fig 2). As the insecticidal impact of the nets becomes more important with increasing coverage, lower levels of repellency enable the parasite to be eliminated from the population at lower coverages (Fig 2). For more repellent nets, ITNs achieve their main impact by diverting mosquitoes from the protected individuals to unprotected ones which consequently receive more infectious bites (Fig 4). Increased repellency offers more personal protection, so that the difference in prevalence between protected and unprotected individuals increases. Neverthless, at sufficiently high coverage increasing repellency also increases prevalence on *protected* individuals (unless repellency is close to perfect) (Fig 4b). The personal protection effect offered by repellent ITN will therefore be most important in a context of low ITN coverage, where diverted mosquitoes find enough alternative hosts and are therefore unlikely to find their way back to the ITN user. Many of the other conclusions are intuitively more obvious. For example, when mosquitoes are zoophilic (Fig 2b), repellency can eliminate mosquitoes at lower coverage than when mosquitoes are strongly anthropophilic because infectious mosquitoes may be diverted to animals (Fig 2a). These patterns are qualitatively similar for highly anthropophilic mosquitoes (Fig 2a) and for mosquitoes that bite humans only rarely (Fig 2b), although of course prevalence is lower for the latter.

**Fig 5:**
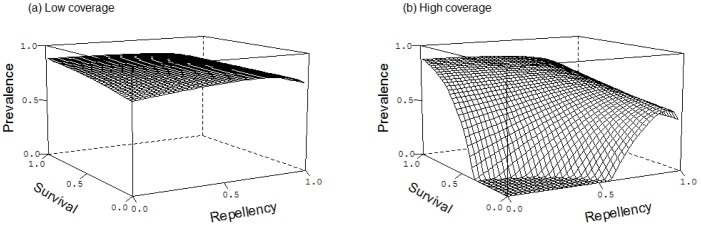
The effects of repellency and probability of surviving the exposure to the insecticide on malaria prevalence. The epidemiological equilibrium prevalence is shown for low ITN coverage (*ϕ* = 0.2) and high ITN coverage (*ϕ* = 0.7). Other parameters are given in Table 1.

The impact of the insecticidal action reflects the role of repellency in being coverage-dependent. At weak repellency and high coverage, increasing insecticidal action (i.e. reducing the probability that mosquitoes survive their bite) strongly reduces prevalence in protected and unprotected individuals (Fig 5b). As survival increases, so does prevalence. With strong repellency, however, the insectidal action of the ITN almost disappears, as most mosquitoes do not contact the ITN. As shown in Fig 2, high repellency also leads to high prevalence because the mosquitoes are diverted to unprotected (and also to protected) individuals. At low coverage the community-wide insecticidal benefit of ITNs is low because few mosquitoes encounter the insecticide, i.e. neither the insecticidal nor the repellenct action has a large impact on total malaria prevalence in the population (Fig 5a).

## Discussion

Insecticide-treated bed nets protect individuals against malaria by blocking and repelling mosquitoes, and they protect the community by killing mosquitoes. The repellent and the insecticidal impacts of the insecticide on control, however, oppose each other: nets that are more repellent reduce the number of mosquitoes that are exposed to the insecticide and therefore kill fewer mosquitoes. As a consequence, our model, which combines the two impacts by merging the biting behaviour of mosquitoes with the epidemiology of malaria, predicts a conflict between individual and community effects: although increasing repellency provides better personal protection, it reduces the community-wide benefit of insecticides and increases the prevalence in the community above the level that could be achieved with non-repellent insecticides. Therefore, at the community level, repellency may be detrimental for the control of malaria.

Specifically, the opposing forces of the repellent and the insecticidal actions lead to three other important predictions: (i)Higher levels of repellency offer better protection to bed net users, but divert more mosquitoes to unprotected people, and thus increase their risk of infection. Stronger repellency therefore leads to greater difference in prevalence between protected and unprotected people. More surprisingly, at a given coverage, stronger repellency also increases the prevalence in *protected* people. The reason is that repelled mosquitoes are diverted not only to animals and unprotected individuals, but also to other ITN-users. Unless repellency is perfect, some of these will penetrate the defence of the net and bite the person sleeping under it. If coverage is sufficient (i.e. if a sufficient number of mosquitoes are diverted from all the ITNs), the repellency of a given net will be outweighed by the increased number of mosquitoes attempting to bite. Exactly the same increase of infection risk in ITN users was observed in the model proposed by Killeen et al. [17].

(ii) If repellency is weak, increasing the coverage of ITNs kills more mosquitoes, thereby decreasing transmission and offering community-wide protection of people with and without a net. As most of the protection is due to the insecticidal action of the insecticide, the community effect dominates so that there is little difference of the prevalence between the two groups. If, however, repellency is strong, the effect of the ITNs is dominated by personal protection. Unprotected individuals therefore are at risk from the diverted mosquitoes, and this risk increases with coverage unless coverage is so high that the unprotected individuals benefit from a herd-effect. (iii) At low coverage or high repellency, few mosquitoes encounter the insecticide. Therefore, in these situations losing the insecticidal action by the evolution of resistance has less impact. Rather, we would expect that evolution would favor behavioural changes of the mosquito, for example by biting at a time when people are still outdoors, as a response to the use of ITNs. Only when coverage is high and repellency low will the evolution of resistance substantially increase the prevalence of disease. In this situation, we would expect strong evolutionary pressure for resistance. Thus, repellency underlies a second public health conflict: between short-term and long-term success. Although stronger repellency may increase prevalence, it will delay the evolution of insecticide resistance, as we show in a different paper [33]. There is however a distinct possibility that genetic resistance to insecticides is genetically linked to the behavioural trait of failing to be repelled by ITNs, as potentially observed in *A. gambiae* [34]. If mosquitoes fail to be repelled and killed by the insecticide, ITNs are reduced to their protective feature of establishing a physical obstacle between human and mosquito and, though repellency will no longer cause a conflict between users and non-users, ITNs will also lose any community-protective effect.

(iv) The general patterns are only slightly affected by the level of zoophily, although, of course, zoophily decreases the overall risk of infection. While repellency diverts many mosquitoes to animals, some will be diverted to humans (whether protected or not). Therefore the predictions are qualitatively similar, though the effects are less strong for zoophilic than for anthropophilic mosquitoes. Similarly, as long as vectors have some degree of zoophily (which may also be determined by the host composition in a given transmission setting), repelled mosquitoes may be diverted to animals. Hence the more zoophilic a vector is, the less conflict is introduced by a repellency (graphs not shown).

It has been suggested that the impacts of ITNs and of vaccines are comparable, for they both lead to a herd effect, where protecting some individuals can protect non-users by reducing the rate of transmission [19]. Our model suggests that this can, indeed, be the case if the ITN is only weakly repellent. There is, however, a crucial way of how ITNs differ from vaccines: vaccinated hosts do not divert pathogens, whereas hosts sleeping under an ITN divert mosquitoes to unprotected individuals. Our model shows that this difference has important consequences: if repellency is strong, personal protection can lead to higher prevalence in unprotected individuals. In this context, the function of ITNs act differently to vaccines: whereas a vaccine provides personal protection and protects surrounding unvaccinated people, ITNs provide personal protection but could expose surrounding unprotected people at a higher risk. Thus, ITNs can lead to a clear conflict between individual and community effects.

Our results corroborate several other models that predict that the repellent action of ITNs can increase the prevalence in unprotected individuals e.g. [3, 4, 16, 17]. We agree with Killeen et al [19] who found that for the highly anthropophilic mosquito *Anopheles gambiae* relative exposure to non-users stays high across the whole range of repellency for a given coverage. Our results, however, differ in several important respects, partly because we considered a greater range of parameter values (e.g. coverage levels), and partly because we allowed an epidemiological feedback between the mosquitoes’ behaviour and the risk that they become infected.

Whereas Killeen et al [19] report an increase in personal protection with repellency while keeping the coverage constant at 75%, we found that repellency only improved personal protection at low coverage levels, as discussed in point (ii). Though, the model of [17] also shows that personal protection degrades at high repellency levels, their model does not highlight the effect of coverage and how it interacts with repellency as they examine their model only at a fixed coverage of 80%. Like [16, 17, 19], we find that the insecticidal property of ITNs is the most important determinant of the community effect of ITNs. In contrast to the earlier studies, however, we suggest that this is the case only when repellency is low and coverage is high, i.e. where the impact of the ITNs is dominated by the insecticidal action rather than the repellency. Most field studies suggest a positive community-wide effect of ITNs [12, 13, 35–37]. Most of these have been conducted in communities with a very high coverage of bed nets (ranging from 70% to near complete coverage), i.e. in conditions where our models also predict a strong community effect. Even in such conditions, bed net users typically have prevalences which are around 30%-40% lower than in non-users [6, 38, 39].

In field settings it is difficult to test which feature of the net is responsible for decreased prevalence in the population. Studies that compare communities using treated and untreated nets could provide some proxy for the effect of the insecticide. While there is some support for the superiority of ITN over untreated bed nets e.g. [11, 13, 35, 40]—and thus for the superiority of the combined insecticidal and repellent actions of the ITNs—research on the effect of ITN repellency alone has given mixed results. Repellency is still widely seen as a desirable feature of vector control; its use in clothing, topical repellents, ITNs and area repellents is a well-established protective measure. The evidence that repellents provide efficient personal protection is compelling [41, 42]. Following this trend, there is a body of research that considers the application of additional repellents to ITNs but so far it remains unclear whether it offers any benefits for malaria control. A model proposed by Kiszewski et al. [43], for example, predicts that an efficient repellent would reduce malaria infection to a level lower to that achieved by ITNs. They assume, however, that the biting rate per untreated person stays constant, which (as we argue here) is unlikely to be the case in particular in areas where mosquitoes are highly anthropophilic. In contrast, a recent field study has shown that using topical repellents are probably overpowered by the much stronger repellent effect of the ITN, therefore making them superfluous [44]. The idea that repellency increases the mosquito biting rate on non-users has received mixed support from field studies. Hewitt et al. [45] finds that ITN-applied repellent is strong enough to protect nearby unprotected people in a house. In contrast, Moore et al [15] find that unprotected people sitting one meter away from people wearing topical repellent experience up to 36% more mosquito landings. It is therefore unclear at which spatial scale repellency operates and it seems to depend strongly on the type of intervention with freshly impregnated or new ITNs offering a larger repellency radius than ITNs whose impregnation has worn off or topical repellents of their own. Repellency has also received attention under “push-pull” approaches of malaria control, which have are claimed to offer strong potential as an intervention if deployed over a wider area [46]. Our model suggests that an ineffective push-pull system, i.e. where the pushing component is more important than the trapping component, could potentially put at risk unprotected people, but the latter depends a lot on the vector species and therefore on its feeding preferences [47, 48]. However, the possibility remains that the push-pull approach could be used to partly offset the “excess” mosquitoes that are repelled by ITNs, especially when coverage is high. Regarding the potential negative effect of repellency on community-protection level we argue that it should be the subject of more extensive field research to find out first whether the phenomenon does take place in real transmission settings and second, if so, how to off-set it in, a push-pull system only being one example. It is also important to keep track of the actual transmission context, especially about the mosquito community composition and the feeding habits of the different species because these are paramount to choosing the optimal intervention strategy [1]. The latter becomes clear in the model proposed by [49] who focus on more zoophilic mosquitoes, which are the most important malaria vectors outside Africa. If transmission is dominated by zoophilic mosquitoes it becomes irrelevant if mosquitoes are targeted by purely toxic or purely repellent compounds, but our model suggests that repellency is still counter-productive at the community level.

Another aspect not occurring in our model but potentially being important in real life transmission settings is the development of adaptive immunity to malaria, which is reliant on repeated exposure to infectious mosquito bites [50, 51]. As ITNs precisely prevent infectious bites, concerns have been raised that this may result in the delayed acquisition of natural protective immunity and thereby lead to an increase of infection in the long term [52, 53]. Temporarily acquired immunity has been integrated in a number of other mathematical models of malaria e.g. [2, 54, 55] but has not been considered under the original formulation of the Ross-Macdonald model, on which our model is based. However, acquired immunity is loosely defined in malaria and most often designates the situation where the a person has developed some resistance against symptoms but still sustains and transmits parasites [50, 56, 57]. Thus, this subpopulation is still captured here by modelling malaria prevalence rather than disease episodes. Finally, it is important to recognize that the personal protection provided by ITN repellency in case of high indoor feeding may be a significant motivation factor for using it, hence leading to higher coverage rates, which in turn have much a greater effect on prevalence than repellency. Thus, although repellency may be detrimental for the control of malaria, its impact on coverage is likely to be beneficial. We argue by no means against ITNs as an intervention strategy: indeed our model shows that whatever the coverage level, the total prevalence of malaria is always reduced. Our model makes formal observations of how the speed at which prevalence is reduced depends on the ITNs properties and how those properties may have opposing effects at different coverage levels. The finding that malaria elimination is more easily achieved with low repellency levels provide a potential tool to the design an “end-game strategy”, a commonly discussed theme in infectious disease control [58, 59]. In summary, our paper highlights that repellent insecticide-treated bed nets introduce a conflict between personal and community protection for malaria control for areas where the main vector is strongly endophilic. Indeed, despite the personal protection offered by repellency, protecting the community would benefit from finding and using insecticides with less repellent action. However, the interactions between personal, epidemiological, evolutionary and social impact of using ITNs are complex, making predictions about the long-term benefits of repellency difficult.
